# Electric and Magnetic Field-Driven Dynamic Structuring for Smart Functional Devices

**DOI:** 10.3390/mi14030661

**Published:** 2023-03-16

**Authors:** Koohee Han

**Affiliations:** Department of Chemical Engineering, Kyungpook National University, Daegu 41566, Republic of Korea; han.koohee@knu.ac.kr

**Keywords:** soft matter, active colloids, directed assembly, dynamic structuring, collective patterns, smart functional devices

## Abstract

The field of soft matter is rapidly growing and pushing the limits of conventional materials science and engineering. Soft matter refers to materials that are easily deformed by thermal fluctuations and external forces, allowing for better adaptation and interaction with the environment. This has opened up opportunities for applications such as stretchable electronics, soft robotics, and microfluidics. In particular, soft matter plays a crucial role in microfluidics, where viscous forces at the microscale pose a challenge to controlling dynamic material behavior and operating functional devices. Field-driven active colloidal systems are a promising model system for building smart functional devices, where dispersed colloidal particles can be activated and controlled by external fields such as magnetic and electric fields. This review focuses on building smart functional devices from field-driven collective patterns, specifically the dynamic structuring of hierarchically ordered structures. These structures self-organize from colloidal building blocks and exhibit reconfigurable collective patterns that can implement smart functions such as shape shifting and self-healing. The review clarifies the basic mechanisms of field-driven particle dynamic behaviors and how particle–particle interactions determine the collective patterns of dynamic structures. Finally, the review concludes by highlighting representative application areas and future directions.

## 1. Introduction

Soft matter is a rapidly growing field that pushes the boundaries of conventional materials science and engineering [1]. As the term suggests, soft matter refers to materials that are easily deformed by thermal fluctuations and external forces. Accordingly, compared to conventional hard materials, soft materials better adapt to and interact with the surrounding environment. Such soft material properties have opened up opportunities for future applications such as stretchable electronics, soft robotics, and microfluidics [2,3,4]. In particular, soft matter plays an increasingly important role in microfluidics. This is largely because, at the microscale, the dominance of viscous forces poses a formidable challenge to controlling the dynamic behaviors of materials and operating functional devices [5,6].

Field-driven active colloidal systems are a promising model system to realize discrete dynamic functions for building smart functional devices at the microscale level [7,8,9]. In field-driven colloidal systems, the dispersed particles can be activated and controlled by external fields, where the activated particles move, interact, assemble, and reconfigure [10,11,12]. In particular, the use of magnetic and electric fields has shown promise to remotely and precisely control a large number of colloidal particles in a programmable way by modulating field parameters such as field direction, amplitude, and frequency [13,14,15]. Recent studies have demonstrated the feasibility of field-driven active colloidal systems to mimic the collective patterns observed in living systems [16,17,18,19].

**Figure 1:**
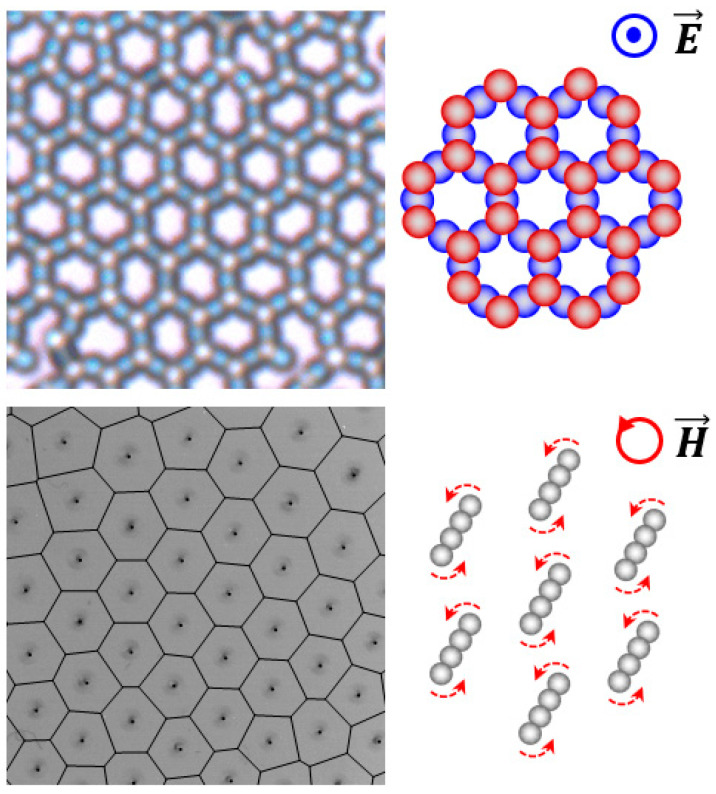
Dynamic structuring of hierarchically ordered structures. (**Top**) Honeycomb-like open structure emergent in a vertical electric field. In the schematic on the right side, the circle and dot with the electric field denote that the field is applied perpendicular to the plane of view. Reproduced with permission [20]. Copyright 2013, American Chemical Society. (**Bottom**) Hexagonal lattice structure emergent in an external in-plane rotating magnetic field. In the schematic on the right side, the dotted red arrows indicate the rotational direction of the multi-particle chains in response to the rotating field. Reproduced with permission [21]. Copyright 2020, American Association for the Advancement of Science.

This review discusses a potential strategy of building smart functional devices from the field-driven collective patterns. The special focus of the collective patterns is on the dynamic structuring of hierarchically ordered structures (Figure 1) [20,21]. Such dynamic structures self-organize from colloidal building blocks and exhibit reconfigurable collective patterns while existing out of equilibrium under external fields. The emergent dynamic structures can implement smart functions such as shape shifting and self-healing by reorganizing their building blocks in reaction to the environment.

In realizing smart devices with tunable functional properties, it is important to identify the interaction–structure–function relationship of the dynamic building blocks. To this end, the review begins with clarifying the basic mechanisms of field-driven particle dynamic behaviors. This is followed by a discussion of how field-driven particle–particle interactions determine the collective patterns of dynamic structures depending on the system design parameters. Then, this review is concluded by highlighting representative application areas and future directions.

## 2. Basic Mechanisms of Field-Driven Particle Behavior

Colloidal particles can exhibit a variety of field-driven dynamic responses when subjected to external fields such as acoustic, optical, magnetic, and electric fields [22,23,24]. This review focuses on colloidal particles powered by magnetic and electric fields, as these energy sources have been found to be suitable to generate collective patterns for the following reasons. First, the application of magnetic and electric fields facilitates the remote supply of energy over long distances so that a large group of particles can be activated simultaneously. Second, the activated particles can be finely controlled by modulating the field parameters such as field amplitude and frequency. Lastly, several different mechanisms of the particle dynamic behaviors can be implemented by engineering key design elements such as the particle design and applied field direction, which will be discussed in detail below.

**Figure 2:**
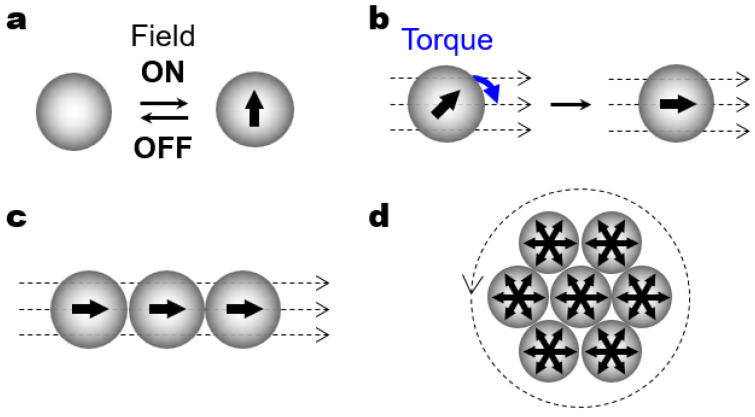
Basic mechanisms of field-driven particle behavior. (**a**) Particle polarization in response to the application of an external (magnetic or electric) field. (**b**) Field-driven torque aligning the induced dipole of the particle along the field direction. The blue arrow represents the alignment torque exerted by the external field. (**c**) The assembly of polarized particles into a linear chain as a result of anisotropic dipolar interaction. (**d**) The assembly of polarized particles into a hexagonal structure as a result of time-averaged isotropic dipolar interaction. The arrows inside the particles indicate the direction of an induced dipole. The dotted arrows outside the particles indicate the direction of external fields.

The particle dynamic behaviors powered by magnetic and electric fields often rely on the effective dipole induced inside colloidal particles. Here, the particles polarize and attain an induced dipole immediately after the application of magnetic or electric field, and they retain the dipole unless the field is removed (Figure 2a). The effective particle polarization requires the discrepancy in polarizability between the particles and surrounding medium [25]. Accordingly, it is necessary for the magnetic polarization to contain magnetic materials in either the particles or the medium. Conversely, the electric polarization is less affected by material selection due to the moderate contrast of electric polarizability between most particles and an aqueous medium.

In a single-particle level, the external fields may exert a torque on the particles with an induced dipole [25,26,27]. When the direction of the external field and the induced dipole inside the particles mismatch, the particles may experience the field-driven torque and align along the direction of an external field (Figure 2b). In a rotating magnetic or electric field, for example, the magnetic or electric torque is present where the particles keep rotating in synchrony with the change in the direction of the external field. In most cases, as long as the field direction does not change, the particles remain aligned along the field direction since the external torque is no longer present.

In a multi-particle level, the charged particles may exhibit directed assembly patterns as directional particle–particle interactions kick in. Despite their different origin, the effective (electric or magnetic) polarization leads to similar dipole–dipole (or dipolar) interactions that can be analyzed based on the point-dipole approximation model [25]. One representative outcome of such field-induced dipolar interactions is dipolar chaining, where polarized particles in a uniaxial field form linear chains of the particles with head-to-tail configuration (Figure 2c). Importantly, the assembled clusters may create non-uniform local fields of high intensity and attract the neighboring particles by dielectrophoretic (or magnetophoretic) forces [28,29]. As a result of synergistic combination of dipolar chaining and dielectrophoretic (or magnetophoretic) forces, multiple linear chains can be further assembled into close-packed crystalline structures under the continuous application of the same field [30,31].

The field-induced dipolar interactions can be more complex in time-dependent multiaxial (e.g., biaxial and triaxial) fields where the field direction and magnitude change periodically in a time-dependent manner [32,33,34]. In time-dependent electromagnetic fields, the polarized particles experience time-averaged dipolar interactions, which may assemble more complex structures than linear chain assemblies. One simple example of such time-dependent fields is an in-plane rotating field that can be generated by orthogonally placed biaxial fields with two sinusoidal signals with a quadrature (90°) phase difference (Figure 2d). Furthermore, a balanced triaxial magnetic field can be created by adding a *z*-axis vertical field to an *xy* in-plane rotating field. Such time-dependent multiaxial fields could be used as an assembly tool for hierarchically ordered dynamic structures.

## 3. Field-Driven Collective Patterns

Self-assembly is a bottom-up approach by which macroscopic materials form as a result of self-organization of nano- and microscopic building blocks [35,36,37,38]. Two major types of self-assembly are the following: static and dynamic [39,40]. Static assembly often results in structures at global or local equilibrium, fixing the assembled pattern (e.g., hexagonal closed packed crystal structures). Dynamic assembly, in contrast, results in out-of-equilibrium reconfigurable structures that occur only while external energy is continuously supplied [41]. Both static and dynamic assembly can be implemented in field-driven colloidal systems.

This review focuses on the dynamic assembly of hierarchically ordered dynamic architectures in external fields. While the hierarchically ordered stable structures of these field-driven dynamic architectures may resemble static self-assembled structures formed without external fields, such as hexagonal closed or non-closed packed crystal structures [36], there is a significant difference between them. Unlike the fixed pattern resulting from static assembly at global equilibrium, these dynamic architectures exist out of equilibrium while continuously dissipating energy from the external fields to retain their hierarchical ordering. Their out-of-equilibrium nature allows for building complex hierarchical ordering that is not easily accessible by static self-assembly and even transforming their hierarchical ordering by reorganizing their building blocks in reaction to their environment and the external fields. However, once the external fields are removed, their hierarchical ordering may disappear.

### 3.1. Electric Field-Driven Dynamic Architectures

**Figure 3:**
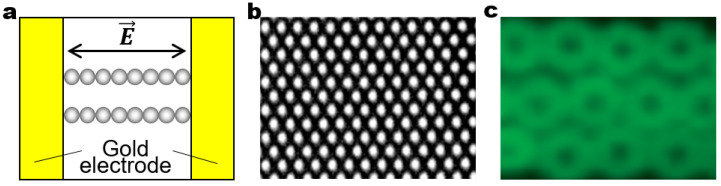
Field-driven dynamic structuring in a planar electric field. (**a**) Schematic of the experimental setup for a planar electric field. An AC electric field is applied between gold electrodes. Upon application of the field, dispersed particles self-assemble into linear chains aligned parallel in the field direction. The linear chains can be further assembled into 2D colloidal crystal structures from (**b**) 1.4 μm spherical (Reproduced with permission [42]; Copyright 2004, American Chemical Society), (**c**) 2.5 μm hexagonal (Reproduced with permission [43]; Copyright 2008, American Chemical Society).

One of the simplest realizations of dynamic architectures can be achieved by the dielectrophoretic (DEP) assembly of particles. For example, spherical particles can be assembled into close-packed crystalline structures in a planar alternating current (AC) electric field (Figure 3a). The field-induced dipolar interactions assemble multiple linear chains, but dipolar attraction may not be strong enough to crystallize the particles. The DEP forces drive the particles to be attracted to regions of high field intensity that form on the periphery of assembled particles and on the bottom surface of the substrate between the coplanar electrodes. A combination of dipolar chaining and DEP forces facilitates the crystallization of the particles at the bottom surface of the substrate (Figure 3b) [42]. The same principle of DEP assembly can be extended to create dynamic architectures with tailored structures by simply replacing the spherical particles with more complex building blocks such as particles with anisotropy in shape and/or polarizability. The examples include the DEP assembly of hexanuts (Figure 3c), rods, discs, ellipses, squares, and rectangles [43,44,45].

**Figure 4:**
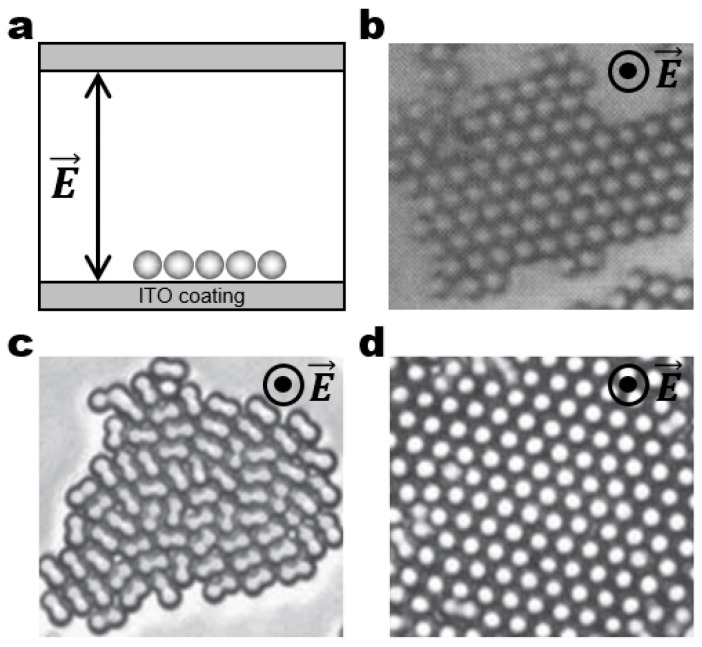
Field-driven dynamic structuring in a vertical electric field with low frequency and high salt concentrations. (**a**) Schematic of the experimental setup for a vertical AC electric field along the *z*-direction. An AC electric field is applied between indium tin oxide (ITO)-coated electrodes. Upon application of the low-frequency field, the dispersed particles adjacent to the bottom electrode experience EHD attraction, leading to their self-assembly. (**b**) Assembly of 2 μm polystyrene particles into crystalline structures. Reproduced with permission [48]. Copyright 1996, American Association for the Advancement of Science. (**c**,**d**) Assembly of symmetric dimers (~1.5 μm × ~2.8 μm) into close-packed structures. Reproduced with permission [49]. Copyright 2012, Wiley. (**c**) The close-packed structure of lying dimers at the field frequency of ~0.6 kHz. The dominant hydrodynamic torques align the dimers parallel to the bottom surface. (**d**) The close-packed structure of standing dimers at the field frequency of ~6 kHz. Each dimer appears as a circle because of the dominant electric torque aligning the long axis of the dimers along the field direction. The circle and dot with the electric field denote that the field is applied perpendicular to the plane of view in (**b**–**d**).

**Figure 5:**
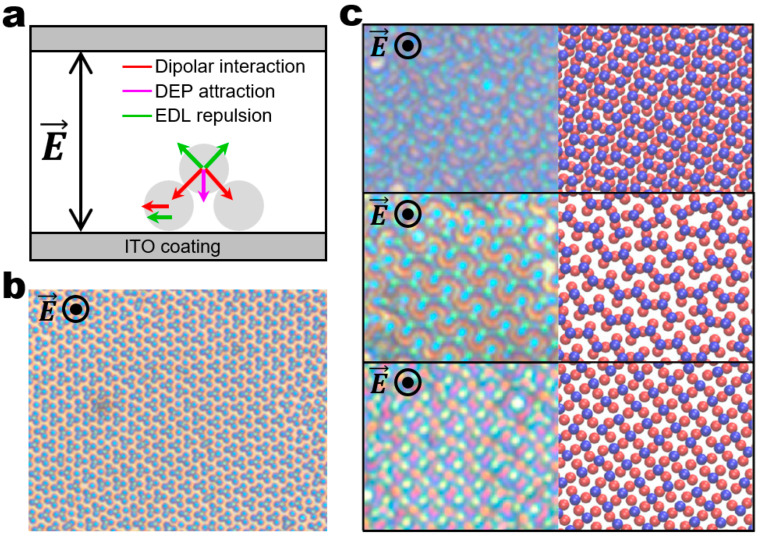
Field-driven dynamic structuring in a vertical electric field with low frequency and low salt concentrations. (**a**) Schematic illustrating the key forces acting on the system. Reproduced with permission [20]. Copyright 2013, American Chemical Society. (**b**) A large array of tetramers assembled from 1.2 μm spherical polystyrene particles. Scale bar: 10 μm. Reproduced with permission [20]. Copyright 2013, American Chemical Society. (**c**) Experimental (left) and simulation (right) results of rectangular bands (top), zig-zag stripes (middle), a sigma lattice (bottom) assembled from 1.2 μm spherical polystyrene particles. Reproduced with permission [50]. Copyright 2021, American Chemical Society. The circle and dot with the electric field denote that the field is applied perpendicular to the plane of view in (**b**,**c**).

**Figure 6:**
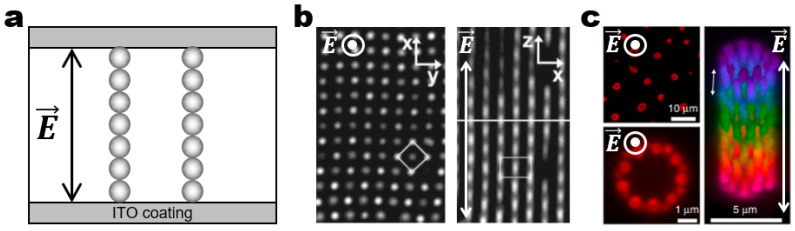
Field-driven dynamic structuring in a high-frequency vertical electric field. (**a**) Schematic of the experimental setup for a vertical AC electric field along the *z*-direction. Upon application of the high-frequency field, the dispersed particles experience strong dipolar attractions and electric torques, leading to the formation of linear chains aligned parallel to the field direction. (**b**) Assembly of 0.525 μm silica spheres into body-centered tetragonal (BCT) crystal structures. (left) The *xy*-plane view illustrating the BCT lattice. (right) The *xz*-plane view illustrating the assembled linear chains aligned parallel to the field direction. Reproduced with permission [52]. Copyright 2000, American Institute of Physics. (**c**) Assembly of ellipsoidal particles into tubular structures. (left) The *xy*-plane views illustrating the empty circle inside the assembled tubular structures. (right) The representative tubular structure aligned along the *z*-axis. Reproduced with permission [54]. Copyright 2014, Nature Publishing Group. The circle and dot with the electric field denote that the field is applied perpendicular to the plane of view in (**b**,**c**).

A wider variety of electric field-driven dynamic architectures can emerge when building blocks are sandwiched between conducting electrodes (Figure 4, Figure 5 and Figure 6). One big advantage of using a vertical AC electric field over using the planar field is that the building blocks are not limited to 2D, but they can construct 3D structures due to the presence of electric torques and dipolar interactions acting perpendicularly. Additionally, in this electrode configuration, a conducting substrate is placed underneath dispersed building blocks such that electrohydrodynamic (EHD) flows can emerge from the interaction between polarized particles and the free ions of the adjacent conducting electrode [46,47]. In this system, the field frequency, field amplitude, and salt concentration are the key parameters to determine the assembly patterns of dynamic architectures by tuning the balance between DEP forces, dipolar forces, EHD flows, and electric torques. 

In a vertical AC electric field with low field frequencies and high salt concentrations, particles can be assembled into crystalline ordered structures like the ones by the DEP assembly in the planar field (Figure 4a). The fundamental difference is that, instead of DEP attraction, EHD flows play a dominant role in crystallizing the building blocks into dynamic architectures by attracting the neighboring particles to each other. Such EHD attraction often results in the organization of particles into close-packed crystalline structures (Figure 4b) [48]. The assembly principle using EHD attraction can be extended to any polarizable particles. Ning Wu and co-workers reported the EHD assembly of dimer particles into close-packed crystalline structures (Figure 4c,d) [49]. They demonstrated the on-demand transition of the assembly patterns by tuning the field frequency and modulating the balance between the electric and hydrodynamic torques (Figure 4c,d). More specifically, close-packed lying dimers form when hydrodynamic torques induced by EHD flows dominate (Figure 4c), whereas close-packed standing dimers form when perpendicular electric torques dominate (Figure 4d). 

The dynamic architectures discussed so far predominantly exhibit close-packed ordering. The tuning of field amplitude can be used to render crystalline ordered structures non-close-packed. The higher field amplitude leads to the stronger dipolar repulsion between the neighboring particles since the dipole of each particle aligns along the field direction. The balance between the EHD attraction and dipolar repulsion determines the equilibrium interparticle spacing within non-close-packed ordering. Another route of creating non-close-packed lattices is to change salt concentrations. At lower salt concentrations, the EHD attraction may not be strong enough to bring the particles closely together since the velocity of EHD flows is proportional to the density of free charges. At lower salt concentrations, however, each particle may have a larger Debye length, which prevents the neighboring particles from close-packing due to stronger electric double-layer (EDL) repulsion. Ning Wu, David T. Wu, and co-workers demonstrated the directed assembly of spherical particles into non-close-packed honeycomb lattices in a vertical AC electric field with low field frequency and low salt concentrations by achieving a fine balance between the dipolar interaction, DEP attraction, and EDL repulsion (Figure 5a,b) [20]. Later, they further explored the system based on Monte Carlo simulations that successfully capture the experimental results and construct comprehensive phase diagrams (Figure 5c) [50]. In addition to that, their simulations identified several new phases including rectangular bands, zig-zag stripes, and a sigma lattice, which were then confirmed by experiments. 

In a vertical AC electric field with high field frequencies, polarized particles assemble into long linear chains and columns aligned parallel to the field direction as dipolar attractions and electric torques dominate over the other forces (Figure 6a) [51]. If the dipolar attractions are not strong enough compared to gravitational and thermal energies at a low-field amplitude, the particles may not form 3D structure but just sediment to the bottom. However, at a high-field amplitude, the particles may experience sufficiently strong dipolar attractions leading to 3D assemblies whose structures strongly depend on the particle volume fraction. The dominant structures are chains, sheets, and 3D crystalline structures at a low, intermediate, and high volume fraction of the particles, respectively (Figure 6b) [52]. Yethiraj and van Blaaderen considered the softness effects of interparticle interactions in these model systems by changing the salt concentration in the solvent [53]. On this basis, they demonstrated comprehensive phase diagrams depending on the field amplitude, particle volume fraction, and salt concentrations that accounts for the fine balance between dipolar attraction and charge repulsion. These principles have proven to be extended to the directed assembly of rod-like particles into 3D plastic crystals and glasses. Schurtenberger and co-workers demonstrated the field-directed tubular self-assembly of ellipsoidal particles (Figure 6c) [54]. Their combined experimental and simulation study revealed that tube structures are energetically favorable than sheet structures because of the uncompensated charge on the sheet edges. Later, they performed more comprehensive Monte Carlo simulations to illustrate the importance of shape anisotropy in assembled structures [55]. More specifically, they observed that ellipsoidal particles favor 2D sheets/tubes and 3D crystalline structures at high and low aspect ratios, respectively.

**Figure 7:**
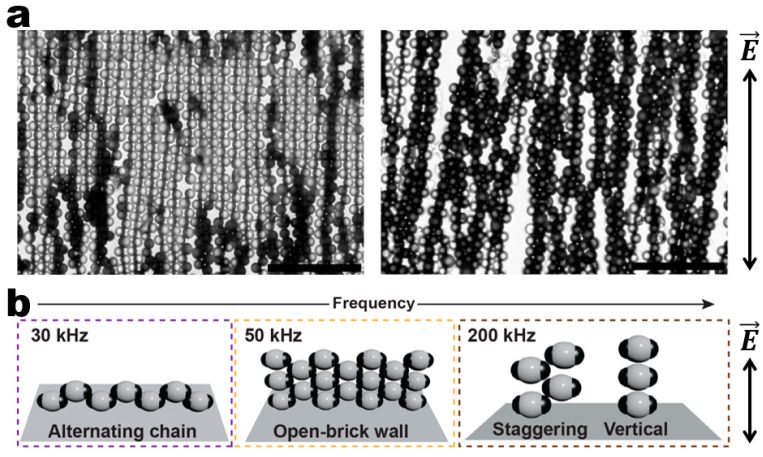
Field-driven dynamic structuring of Janus and patchy particles. (**a**) Assembly of metallo-dielectric Janus particles into (left) staggered chains and (right) 3D bundles in a planar AC electric field. Scale bars: 50 μm. Reproduced with permission [56]. Copyright 2008, American Chemical Society. (**b**) Frequency-dependent dynamic structuring of metallo-dielectric patchy particles [58]. Copyright 2021, American Chemical Society.

A higher level of ordering complexity in assembled architectures can be introduced by using complex particles (Figure 7). The most representative example is the field-directed assembly of metallo-dielectric Janus particles pioneered by Velev and co-workers (Figure 7a) [56]. They reported the directed assembly of Janus particles into regular/staggered chains, 2D crystalline structures, and 3D bundles in a planar AC electric field as well as phase transitions between these various assemblies depending on the field frequency and amplitude. Later, in collaboration with the group of Kretchmar, they investigated the principles of programmed assembly by using more complex metallo-dielectric patchy particles [57]. These particles have proven to assemble into 2D percolated networks and lattices with unusual symmetry through pre-programmed interactions dependent on the patch size, number, and orientation. Wang and co-workers further developed this strategy by investigating the programmed assembly of metallo-dielectric particles with central dielectric matrix and two metallic patches in a vertical AC electric field (Figure 7b) [58]. They demonstrated that these patchy particles can be assembled into a rich diversity of reconfigurable assembled structures including two-layer alternating chains, open-brick walls, staggering stacks, and vertical chains dictated by the particle patches, field frequency and medium conditions. 

### 3.2. Magnetic Field-Driven Dynamic Architectures

In the context of requiring effective polarization of particles, magnetic field-driven assembly is similar in principle to electric field-driven assembly. Indeed, magnetic and electric field-driven assembly can generate similar assemblies. However, the magnetic and electric polarization have fundamentally different origins. The most critical difference is that magnetic materials are essential for magnetic polarization. The most common magnetic particles susceptible to magnetic polarization are superparamagnetic (iron oxide) and ferromagnetic (iron, nickel, and cobalt) materials. The mechanisms of building dynamic architectures from these magnetic particles are often governed by dipolar interactions and magnetic torques [59], as discussed in detail below.

**Figure 8:**
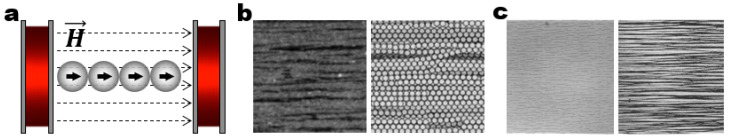
Field-driven dynamic structuring in a uniaxial magnetic electric field. (**a**) Schematic of the experimental setup for a unidirectional magnetic field. Upon application of the field, dispersed particles self-assemble into linear chains aligned parallel in the field direction. (**b**) Experimental (left) and simulation (right) results illustrating the chain-like structures assembled along the field direction. Fe particles of size 4 μm were used in the experiment. Reproduced with permission [60]. Copyright 2000, American Physical Society. (**c**) Structural evolution in the assembly of 1.5 μm superparamagnetic particles. (left) Assembly of scatter linear chains at low volume fraction. (right) Emergence of fibrous structures as a result of growth and coalescence of the chains at high volume fraction. Reproduced with permission [61]. Copyright 1992, Elsevier.

In the simplest case, a uniaxial magnetic field drives the assembly of magnetic particles into linear chains. Because of dipolar chaining, the assembled chains may align parallel to the field direction with the head-to-tail configuration (Figure 8a). These chains may aggregate in a lateral direction and form network-like structures (Figure 8b) [60]. However, the lateral aggregation of the chains is a diffusion-limited process, so a large number of particles are required for the network formation. Fermigier and Gast reported the structural evolution of a suspension of superparamagnetic particles in a static magnetic field (Figure 8c) [61]. They investigated the effects of particle concentration and dipolar interaction strength on the formation of interconnected network and fibrous structures. Following the studies by Promislow and Gast [62,63], Furst and co-workers employed an approach of pulsing the static magnetic field at a given frequency to generate more various network structures [64]. The basic idea is to tune the pulse frequency with respect to the characteristic relaxation time of the suspension. A low pulse frequency, for example, gives the suspension enough time to relax and reach lower energy states. A high pulse frequency, on the other hand, does not allow enough relaxation time for the suspension, causing the percolated networks to remain kinetically arrested. Recently, they demonstrated more sophisticated control over the network structures by varying the duty cycle of the pulsed magnetic field [65]. More specifically, they built an expanded phase diagram that includes columnar, ellipsoidal, percolated, and perpendicular structures by varying the pulse frequency and the fraction of field-on duration in each pulse cycle. Another route of generating interconnected network structures is to use a biaxial magnetic field. Maekawa and co-workers reported the formation of network structures interconnected by T, L, and criss-cross junctions by combining direct current (DC) and AC magnetic fields in orthogonal directions [66].

**Figure 9:**
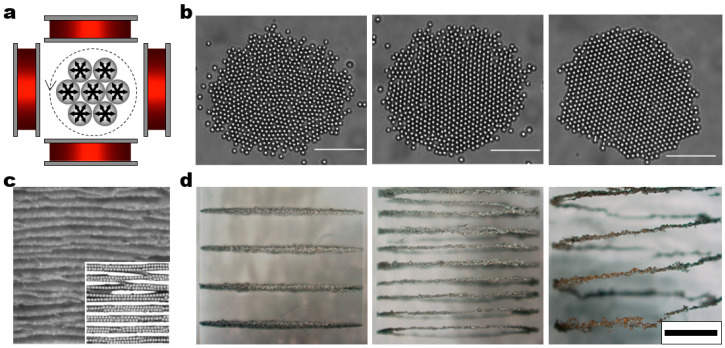
Field-driven dynamic structuring in a high-frequency rotating magnetic field. (**a**) Schematic of the experimental setup for a biaxial rotating magnetic field. Upon application of the field, dispersed particles self-assemble as a result of time-averaged isotropic dipolar interactions. (**b**) The phase behavior of assembled structures of 1.1 μm carboxyl-coated paramagnetic particles depending on the field amplitude: (left) disordered fluid-like phase at the field amplitude of 8.5 Gauss, (middle) quasi-crystalline phase at the field amplitude of 9 Gauss, and (right) ordered crystal phase at the field amplitude of 11 Gauss. Scale bars: 10 μm. Reproduced with permission [68]. Copyright 2018, American Physical Society. (**c**) Assembly of 4 μm Fe particles into 3D multi-layered parallel sheet structures. The inset shows the corresponding simulation result. Reproduced with permission [60]. Copyright 2000, American Physical Society. (**d**) Assembly of 80 μm Fe particles into (left) 3D multi-layered parallel plates, (middle) 3D multi-layered parallel rings, and (right) 3D helical coil structures. Scale bar: 2000 μm. Reproduced with permission. Copyright 2017, Wiley [70].

Time-dependent magnetic fields can be employed to tune particle dipolar interactions from anisotropic to isotropic. A rotating field is a specific type of time-dependent field in which two orthogonally positioned biaxial AC fields are applied with a 90° phase difference (Figure 9a). The use of rotating magnetic fields facilitates the crystallization of polarized particles by time-averaged isotropic dipolar interactions. Biswal and co-workers demonstrated that a high-frequency rotating magnetic field (HFRMF) can be used to induce highly tunable, long-range attractive interactions between superparamagnetic particles [67]. They derived an analytical model equation that accounts for time-averaged interactions in HFRMF. According to this model equation, the interactions between the particles are isotropic and independent of rotation angle. This is because, at a high enough rotational frequency, there is no effective particle rotation as frictional torques balance out the magnetic torque. They showed that gas, liquid, and crystal phases in 2D can be achieved by changing the field amplitude and tuning the strength of long-range attractive interactions. Later, they revealed the tunable interfacial stiffness of these particle assemblies by examining loosely-packed disordered and close-packed ordered structures at different field amplitudes (Figure 9b) [68]. Following this, they looked at a larger system composed of polycrystalline sheets and investigated the effects of interfacial shear at grain boundaries, which brings a new insight into the understanding of grain boundary dynamics [69].

Beyond 2D crystallization, time-averaged dipolar interactions in a rotating magnetic field can be extended to produce multi-layered 3D sheet structures. The underlying principle is that particle–particle interactions are attractive in the same plane of rotation but repulsive along the axis of rotation. So, in principle, in one plane of rotation, dominant dipolar attraction between the particles results in a 2D crystalline sheet, as discussed above. However, in 3D, multi-layered parallel sheet structures may emerge as a result of dipolar repulsion along the axis of rotation. Martin and co-workers realized these sheet-like particle assemblies in both experiments and simulations (Figure 9c) [60]. Actually, in order to create a rotating magnetic field, the sample cell was directly rotated in a static magnetic field instead of using a conventional rotating magnetic field setup composed of electromagnetic coils. However, the experimental results from this simplified model system agreed well with the simulations. They demonstrated the concept of magnetic field-structured composites by preserving the 3D-assembled sheet structures in an epoxy resin. Later, Grzybowski and co-workers made use of this system to generate more complex sheet-like structures (Figure 9d) [70]. More specifically, they diversified the assembled sheet structures from arrays of parallel plates to rings and helices by subjecting a rotating sample cell to more various static magnetic field configurations.

**Figure 10:**
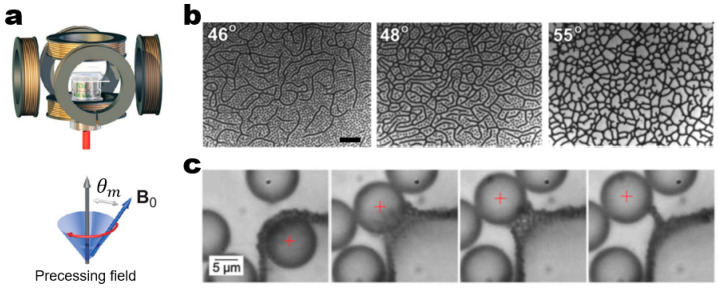
Field-driven dynamic structuring in a precessing magnetic field. (**a**) Schematic of the experimental setup for a triaxial magnetic field: (top) Schematic illustrating a set of three orthogonal pairs of Helmholtz coils that can be used to generate a triaxial magnetic field. (bottom) Schematic representation of a precessing field where $\theta_{m}$ indicates the precessing angle. Reproduced with permission [71]. Copyright 2009, American Physical Society. (**b**) Types of percolated network structures assembled from 1.05 μm superparamagnetic particles depending on the precessing angle. The degree of percolation increases with increasing the precessing angle: (left) percolated long chains at the precessing angle of 46°, (middle) percolated networks at the precessing angle of 48°, and (right) froth-like structure at the precessing angle of 55°. Scale bar: 10 μm. Reproduced with permission [74]. Copyright 2014, American Chemical Society. (**c**) The self-healing property of one-particle-thick membrane formed from 1.05 μm superparamagnetic particles. The magic-angle precessing magnetic field drives the consolidation of membranes via the coarsening of network structures. The membrane spontaneously self-heals when a 9 mm silica particle is passed through the membrane by laser tweezers (indicated by the red cross). Reproduced with permission [71]. Copyright 2009, American Physical Society.

A higher level of ordering complexity in assembled network structures can be introduced by using a triaxial magnetic field. The most representative example of triaxial magnetic fields is a precessing magnetic field that can be created by adding a static vertical magnetic field to an in-plane rotating magnetic field (Figure 10a) [71]. Here, the interaction potential in the system is determined by the relative amplitude ratio of the static *z*-component to the rotating *xy*-component, which can be characterized by the precessing angle. For example, with respect to the rotating *xy*-plane, time-averaged dipolar interactions switch from purely attractive at a precessing angle of 90° (i.e., the pronounced *xy*-plane rotating field) to purely repulsive at a precessing angle of 0° (i.e., the pronounced *z*-axis static field). The two-body interaction potential model gives a good estimate of the interaction potential in these two limiting cases. In this simplified model, the total dipolar interaction energy is the sum of all the pairwise interactions. When it comes to intermediate precessing angles, the coupling of these counteracting *xy*- and *z*-components complicates the time-averaged dipolar interactions [72,73]. The complex dipolar interactions in a precessing magnetic field drive concentrated particles to form intriguing network structures in which the degree of percolation depends on the precessing angle (Figure 10b) [74]. Importantly, at the precessing angle of 54.7° which can be referred to as a “magic angle,” a precessing magnetic field facilitates the structural evolution from the growth of short chains to the coarsening of the network structure via Ostwald ripening [75]. The network coarsening ends up formulating one-particle-thick membranes with a self-healing property (Figure 10c) [71]. The formation of these unusual network architectures is mainly attributed to a combination of two-body isotropic and many-body anisotropic interactions. Therefore, the many-body interaction potential model works better than the two-body interaction potential model in estimating the interaction potential in a precessing magnetic field. The complex time-averaged dipolar interactions in a precessing magnetic field can be extended to produce 3D network structures. Martin and co-workers pioneered this approach by utilizing a balanced triaxial magnetic field in which all *xyz*-components have equal field amplitudes [76,77,78]. They demonstrated the important role of the magic angle in forming open network structures in both experiments and simulations.

## 4. Concluding Remark and Future Outlook

Field-driven active colloidal systems offer a versatile platform to assemble colloidal building blocks into dynamic architectures. In field-driven colloidal systems, the building blocks transduce external energy to directionally interact and assemble. The use of electric and magnetic fields, in particular, enables the remote and precise control of a large number of building blocks in a programmable manner. The system design parameters, such as the field direction, frequency, and amplitude, determine the mechanisms of the particle dynamic behaviors. This field-driven assembly strategy has shown promise for building a variety of hierarchical structures, ranging from 1D chains to 3D network structures, as discussed above.

**Figure 11:**
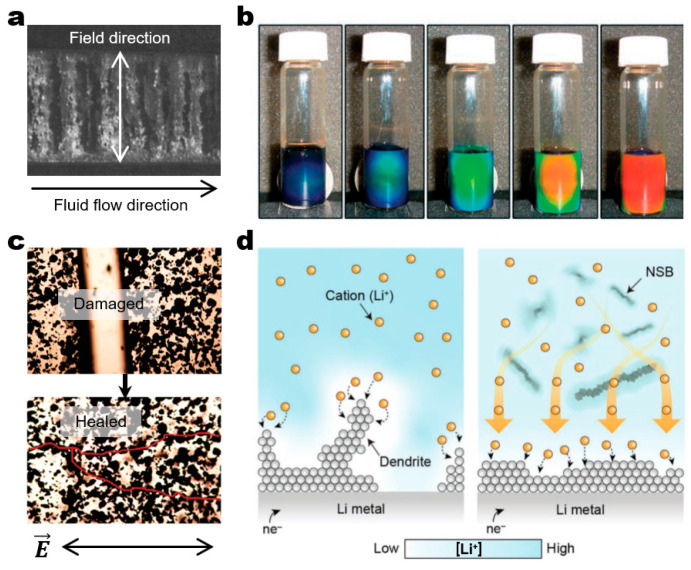
Applications of field-driven dynamic architectures for smart functional devices. (**a**) an electrorheological fluid illustrating the field-driven structuring of fibrous structures from 60 μm spherical silica particles. The formed fibrous structures effectively block the fluid flow, resulting in apparent viscosity. Here, the fluid flow direction is orthogonal to the alignment direction of the fibrous structures. Reproduced with permission [79]. Copyright 1989, Elsevier. (**b**) Magnetic field-responsive photonic crystals of superparamagnetic colloidal nanocrystal clusters, displaying different colors depending on the field amplitude. The distance between the sample and a MdFeB magnet determines the field amplitude and modulate the lattice spacing of the photonic crystals. Reproduced with permission [83]. Copyright 2007, Wiley. (**c**) Electric field-driven self-healing of conductive microwires of eutectic gallium indium (EGaIn) microdroplets. (top) The mechanically damaged circuit. (bottom) The self-healed circuit via DEP assembly of EGaIn microdroplets upon the application of an electric field. The red lines indicate the interconnected conductive pathways. Reproduced with permission [87]. Copyright 2020, Wiley. (**d**) Dendrite-free Li electrodeposition mediated by magnetic nanospinbars (NSB) in a rotating magnetic field. (left) Li dendrite growth without NSBs. (right) The suppression of Li dendrite growth by redistributing Li^+^ flux with NSBs. (bottom) The color bar represents the distribution of Li^+^ concentration. Reproduced with permission [90]. Copyright 2022, Wiley.

These dynamic architectures provide opportunities for a wide range of applications (Figure 11). Their ability to rearrange building blocks is especially useful for building smart devices that need on-demand switchable functions, as exemplified below. First of all, electrorheological and magnetorheological fluids can significantly change their apparent viscosity in response to external fields (Figure 11a) [79]. These fluids include polarizable particles and immiscible insulating fluids. The field-responsive liquid-to-solid phase transition affects the apparent viscosity where the dispersed polarizable particles form percolated chains in a kinetically jammed state perpendicular to the direction of fluid flow [80,81]. It is noteworthy that these systems were the early example of the field-driven assembly that has served as the major inspiration for the growth of the field. Second, photonic crystals can reversibly change their color in response to magnetic fields [82]. The underlying principle is to magnetically modulate the lattice spacing of colloidal crystals, which determines the structural color (Figure 11b) [82,83]. Electric field-driven photonic crystals have also been reported [84,85]. Third, dynamic architectures with field-driven self-healing properties formulate emerging application areas [86,87,88]. For example, field-driven assembly of dispersed conductive nanoparticles can form electrically functional microwires and even rebuild conductive pathways between disconnected electrodes [87,88]. Dickey and co-workers have recently demonstrated the self-healing of stretchable and flexible conductive microwires by combining liquid metals and silicone polymer matrix that may form a basis for the development of novel stretchable electronic devices (Figure 11c) [87]. Last but not least, dynamic spinner arrays have shown promise for microscale transport and mixing applications [89]. Han, Snezhko, and co-workers showed that the coupling of hydrodynamic flows in synchronized spinner lattices in an in-plane rotating magnetic field facilitates enhanced diffusion of passive particles [21]. Lee and co-workers have recently demonstrated that magnetic nanospinbars generate mesoscale turbulence inside a Li||LiNi_0.6_Mn_0.2_Co_0.2_O_2_ (NMC622) cell, effectively redistributing Li^+^ flux and suppressing Li dendrite growth during Li electroplating (Figure 11d) [90].

Dynamic architecturing in field-driven active colloidal systems has many promising future directions. First, multiple field stimuli can be simultaneously used to introduce a higher level of ordering in particle assemblies [91,92,93]. Because of their different origins, magnetic and electric dipoles act independently. On the basis of this principle, Bharti, Velev, and co-workers showed the directed assembly of multidirectionally percolated network structures in concurrent electric and magnetic fields [94]. In addition to the combination of electric and magnetic fields, other types of fields, such as optical fields, can be combined to give rise to additional control of spatial and temporal organization in assembled structures [95,96,97]. Second, complex particles can be used as building blocks beyond simple spherical particles [98,99,100]. The examples are particles with anisotropic shape and/or polarizability [101,102,103]. The use of such complex building blocks can induce more sophisticated multipolar interactions and build assemblies with broad structural diversity [104,105]. The other directions may include the engineering of system design elements such as medium, templates, and interfaces [106,107,108]. It is believed that the rational design and engineering of dynamic architectures could pave the way for the next generation of functional materials [109,110,111].

## Data Availability

No new data were created or analyzed in this study. Data sharing is not applicable to this article.
